# PAMPs and DAMPs as the Bridge Between Periodontitis and Atherosclerosis: The Potential Therapeutic Targets

**DOI:** 10.3389/fcell.2022.856118

**Published:** 2022-02-25

**Authors:** Xuanzhi Zhu, Hanyao Huang, Lei Zhao

**Affiliations:** ^1^ State Key Laboratory of Oral Diseases, Department of Periodontics, National Clinical Research Center for Oral Diseases, West China Hospital of Stomatology, Sichuan University, Chengdu, China; ^2^ State Key Laboratory of Oral Diseases, Department of Oral and Maxillofacial Surgery, National Clinical Research Center for Oral Diseases, West China Hospital of Stomatology, Sichuan University, Chengdu, China

**Keywords:** PAMPs, DAMPs, PRRs, innate immunity, periodontitis, atherosclerosis

## Abstract

Atherosclerosis is a chronic artery disease characterized by plaque formation and vascular inflammation, eventually leading to myocardial infarction and stroke. Innate immunity plays an irreplaceable role in the vascular inflammatory response triggered by chronic infection. Periodontitis is a common chronic disorder that involves oral microbe-related inflammatory bone loss and local destruction of the periodontal ligament and is a risk factor for atherosclerosis. Periodontal pathogens contain numerous pathogen-associated molecular patterns (PAMPs) such as lipopolysaccharide, CpG DNA, and Peptidoglycan, that initiate the inflammatory response of the innate immunity depending on the recognition of pattern-recognition receptors (PRRs) of host cells. The immune-inflammatory response and destruction of the periodontal tissue will produce a large number of damage-associated molecular patterns (DAMPs) such as neutrophil extracellular traps (NETs), high mobility group box 1 (HMGB1), alarmins (S100 protein), and which can further affect the progression of atherosclerosis. Molecular patterns have recently become the therapeutic targets for inflammatory disease, including blocking the interaction between molecular patterns and PRRs and controlling the related signal transduction pathway. This review summarized the research progress of some representative PAMPs and DAMPs as the molecular pathological mechanism bridging periodontitis and atherosclerosis. We also discussed possible ways to prevent serious cardiovascular events in patients with periodontitis and atherosclerosis by targeting molecular patterns.

## 1 Introduction

Cardiovascular disease (CVD), mainly coronary atherosclerotic heart disease, and is the number one cause of premature death in humans (Roth et al., 2020). Chronic infection and the inflammatory response caused by this infection are important risk factors for the formation of atherosclerosis (AS), the primary pathology of CVD (Soehnlein and Libby, 2021). Innate immunity is the host’s first line of defense against pathogenic microorganisms, it plays a vital role in the vascular inflammatory response triggered by chronic infection (Thaiss et al., 2016). Different from adaptive immune response relies on antigen-specific T/B lymphocytes *in vivo* to activate, proliferate, and differentiate into effector cells after receiving antigen stimulation (Chavarria-Smith et al., 2018), the activation of innate immunity depends on the interaction between pattern-recognition receptors (PRRs) of host cells and molecular patterns, such as pathogen-associated molecular patterns (PAMPs), and damage-associated molecular patterns (DAMPs) (Olive, 2012). The pathogen itself or its metabolites together, as PAMPs, constitute a class of molecular patterns involved in the activation of innate immunity. PAMPs are relatively non-specific, highly conserved, pathogenic molecular structures expressed in pathogens, and their products (Mogensen, 2009). DAMPs are a large number of related intracellular proteins or nucleic acids released by necrotic cells at the site of necrosis. DAMPs participate in the occurrence and development of acute and chronic inflammation and are critical factors in the outbreak of acute severe inflammation (Gong et al., 2020).

Periodontitis (PD) is an infectious inflammatory disease with plaque biofilm as the initiating factor. It mainly destroys the supporting tissues around the teeth (including gingiva, periodontal ligament, alveolar bone, and cementum). The microbial dysbiosis and the host immune response jointly promote the progression of PD (Slots, 2017), in which PAMPs and DAMPs are representatives of this process. Representative PD-related PAMPs include lipopolysaccharide (LPS), peptidoglycan (PGN), and DNA sequence containing unmethylated CpG-motif (CpG DNA). LPS and PGN, which are located on the surface of periodontal pathogenic bacteria, can be released after the bacteria are cleared and lysed, accompanied by the release of CpG DNA (Song et al., 2017). The release of PD-related DAMPs, represented by neutrophil extracellular traps (NETs), high mobility group box l (HMGB1) and alarmins (S100A8, S100A9, and S100A12), mainly comes from the ablation and apoptosis of periodontal tissue cells and the activation and rupture of immune cells (Gu and Han, 2020). PAMPs and DAMPs interact with the PRRs in the periodontal tissues. With the persistent activation of the innate immune system by PAMPs and DAMPs, inflammatory responses continuously exist and lead to the destruction of the periodontal tissue (Song et al., 2017; Gu and Han, 2020).

In periodontal tissues, PAMPs are recognized by PRRs and initiate the innate immune response within a short period, such as eliminating pathogens by macrophages and complement. After PRRs recognize PAMPs, neutrophils, T lymphocytes, macrophages, and plasma cells successively infiltrate periodontal tissue; immune cells secrete IL-1β (interleukin-1β), IL-6, TNF-α (tumor necrosis factor-α) or other cytokines which mediate inflammation (Karki and Kanneganti, 2021), promote osteoclast production, and cause periodontal tissue damage (Chen et al., 2021). DAMPs released in PD were also confirmed to capture bacteria and activate inflammation in PD (Gu and Han, 2020).

**FIGURE 1:**
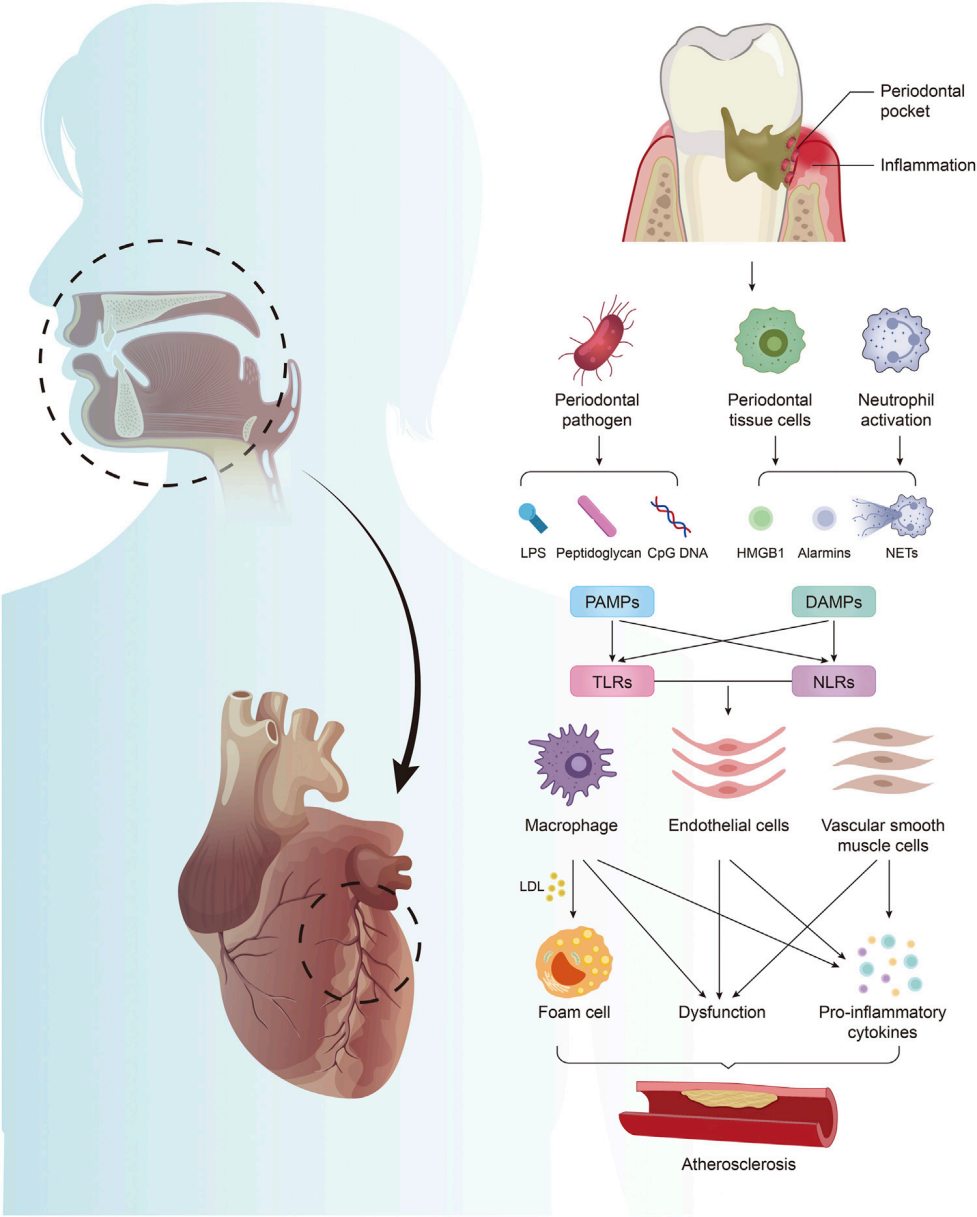
Periodontitis mediates the formation of atherosclerosis by producing PAMPs and DAMPs. Periodontal pathogens produce PAMPs, including LPS, PGN, and CpG DNA. Periodontal infection activates neutrophils to form NETs, which together with HMGB1 and alarmins released by damaged periodontal cells constitute DAMPs. PAMPs and DAMPs activate excessive innate immunity by acting on TLRs and NLRs in arterial tissue, leading to foam cell formation, endothelial cell and vascular smooth muscle cell dysfunction, and promoting the massive release of inflammatory factors, which are involved in the regulation of AS.

PD is regarded as a significant independent risk factor for AS (Holmlund et al., 2017; Sanz et al., 2020). *Porphyromonas gingivalis* (*P. gingivalis*), one primary pathogen of PD (Slots, 2017), can adhere to and invade the arterial vessel wall after entering the bloodstream. By inhibiting the proliferation of endothelial cells, it promotes the adhesion and chemotaxis of monocytes, and activates the inflammatory signaling pathway (Hajishengallis, 2015), eventually leading to vascular endothelial dysfunction (Higashi et al., 2008), aggravating vascular inflammation, and promoting the formation of AS (Gibson et al., 2004). Meanwhile, studies have confirmed that many of these PAMPs and DAMPs related to PD are involved in the progression of AS, and most of them have adverse effects. These substances and the activated innate immunity bridge the gap between PD and AS, and enable us further to understand the relationship between oral diseases and systemic diseases (Figure 1). These substances may also become new targets for treating patients with AS who are aggravated by PD. This review summarizes representative PD-derived PAMPs and DAMPs and their receptors that are most closely related to AS and introduces potential treatments.

## 2 Representative Periodontitis-Related PAMPs and DAMPs and Their Roles in Atherosclerosis

### 2.1 Representative Periodontitis-Related PAMPs

#### 2.1.1 Lipopolysaccharide

LPS is a unique component of the outer membrane of Gram-negative bacteria, composed of lipid A, a short core oligosaccharide and O-antigen. It is also called endotoxin due to its ability to induce a robust inflammatory response. LPS is the most representative virulence factor among periodontal pathogens. Clinical studies have shown that LPS levels were positively correlated with periodontal clinical parameters and inflammatory factors before and after periodontal treatment (Lee et al., 2008; Shaddox et al., 2013).

After the periodontal microbial homeostasis is disrupted, *P. gingivalis* proliferates in large numbers, and excessive proliferation and death cause the release of LPS (Zheng et al., 2021). *P. gingivalis* LPS is recognized by the Toll-like receptor 4 (TLR4) of macrophages and activates the NF-κB and MAPK signaling pathways, thereby inducing the release of inflammatory cytokines (Nativel et al., 2017). These factors can promote the expression of matrix metalloproteinases and osteoclast factors and then destroy soft tissue and bone.

*P. gingivalis* LPS can induce macrophage foam cell formation. It promotes the binding of macrophages to low-density lipoproteins (LDL) and induces macrophages to modify native LDL (Qi et al., 2003). *P. gingivalis* LPS promotes monocyte chemotaxis and adhesion to vascular endothelial cells through Akt and NF-κB signaling pathways (Wang et al., 2020). *P. gingivalis* LPS can also promote the high expression of angiotensin II and IL-6 in vascular endothelial cells and accelerates endothelial dysfunction (Viafara-Garcia et al., 2019). In animal models, *P. gingivalis* LPS increased the secretion of TNF-α from macrophages and up-regulated the expression of endothelial cell adhesion molecules, which aggravated the exacerbated effect of ligature-induced PD on AS (Suh et al., 2019). However, the role of other periodontal pathogenic LPS in AS, such as *Treponema denticola* (*T. denticola*) and *Tannerella forsythia* (*T. forsythia*), is still unknown.

From a broader perspective, PD related low-grade endotoxemia (LGE) causes phenotypic and transcriptional changes in myeloid cell populations that enhance their response to pathogens, a process known as trained immunity (Netea et al., 2020). LPS from periodontal pockets continue to enter the peripheral blood at low levels, activating neutrophil hyperresponsiveness (Vitkov et al., 2021) (The role of neutrophils in this process will be discussed in detail later). Therefore, periodontal pathogenic bacteria-derived LPS represented by *P. gingivalis* LPS and its induced LGE may be the link between PD and AS.

#### 2.1.2 CpG DNA

CpG DNA is a type of DNA sequence with immune activation function containing unmethylated CpG motif, including artificially synthesized oligodeoxynucleotides containing CpG (CpG ODN) and genomic DNA of bacteria, viruses, and invertebrates (Zhou and Deng, 2021). CpG DNA triggers immunostimulatory activity through TLR9 (Ohto et al., 2015). TLR9 is highly expressed in the gingival tissue of PD patients (Narayan et al., 2018), suggesting that CpG DNA could be actively involved in the progression of PD.

It was found *in vitro* that macrophages recognize CpG DNA from periodontal pathogens through TLR9 and then highly express IL-1β and TNF-α to induce osteoclastogenesis (Zou et al., 2002). In addition, TLR9-related autophagy may also be involved in the progression of PD (Wei et al., 2020). However, there are also studies showing that CpG ODN sometimes may positively affect PD. CpG ODNs can promote the proliferation and differentiation of MC3T3 cells in the early stage and up-regulate the expression levels of bone differentiation genes SP7 and OCN (Yu et al., 2020).

AS can associate with PD through CpG DNA. In ApoE^−/-^ mice infected by *P. gingivalis*, alveolar bone resorption and aortic plaque increased significantly (Xuan et al., 2017). The genomic DNA of *P. gingivalis* can be detected in the oral epithelium and the aorta (Velsko et al., 2014). In polymicrobial infection-induced periodontal disease, ApoE^−/-^ mice had enlarged aortic plaques, accumulation of macrophages around the arteries, increased serum cholesterol and triglycerides, while genomic DNA of *P. gingivalis*, *T. denticola*, and *T. forsythia* can be detected in the aorta and liver (Rivera et al., 2013). Intravenous injection of *P. gingivalis* in ApoE^−/-^ mice can also aggravate AS, and the ribosomal DNA of *P. gingivalis* can be detected in the aorta, liver and heart (Li et al., 2002). However, *P. gingivalis* DNA in the periodontal pocket may not be transferred to the heart valve area, causing the aortic valve and mitral valve to degenerate (Radwan-Oczko et al., 2014). Therefore, periodontal pathogens represented by *P. gingivalis* might colonize the arterial wall through blood circulation, and the CpG DNA released after bacterial cell lysis may regulate the development of AS by activating the corresponding TLR9 pathway.

However, the role of CpG DNA/ODN on AS is still controversial. The genetic deletion of the TLR9 gene exacerbated AS lesions in ApoE^−/-^ mice, and the use of CpG ODN 1668 can reduce this effect (Koulis et al., 2014). In other words, CpG ODN and TLR9 may protect the aorta in specific circumstances. Further studies demonstrated that TLR9 plays a negative role in vascular injury (Hirata et al., 2013), and systemic stimulation of TLR9 with high-dose CpG ODN will aggravate the development of AS (Krogmann et al., 2016). There are differences between CpG DNA released in PD and synthetic CpG ODN. The difference in concentration and sequence may lead to changes in the activation of the downstream inflammatory pathway of TLR9 in AS lesions. Therefore, it is necessary to screen PD-related CpG DNA to clarify its role in AS in future studies.

#### 2.1.3 Peptidoglycan

Peptidoglycan (PGN) is a common component of bacterial cell walls. Transcriptional pathways for peptidoglycan synthesis are significantly up-regulated in tongue and subgingival plaque in patients with periodontitis (Yost et al., 2015; Belstrom et al., 2021). PGN can be recognized by TLR2 on the cell membrane and the endogenous NOD1, NOD2, and NLRP3. The expression of NOD1 and NOD2 can be detected in human periodontal ligament cells (hPDLC). Under the stimulation of PGN, the production of IL-6 and IL-8 in hPDLC increased, and the NF-κB and MAPK signaling pathways were activated (Jeon et al., 2012). Injecting PGN into the gums of mice can induce osteoclastogenesis, and TLR2, NOD1, and NOD2 are activated (Kishimoto et al., 2012). N-acetylglucosamine can be recognized by the glycolytic enzyme hexokinase in the cytoplasm and subsequently activate the NLRP3 inflammasome, which may be involved in the progression of PD (Wolf et al., 2016). In summary, periodontal pathogens may activate excessive innate immunity through PGN, an exogenous pathogen-associated molecular pattern, and cause PD. The destruction of periodontal tissue damages the barrier function of the oral mucosa. The decisive invasion and migration capabilities of periodontal pathogens make it enter the circulation and spread the PGN in the cell wall to the cardiovascular system.

Although there are few studies on the relation between periodontal PGN and AS, as a ubiquitous substance in bacteria, PGN produced by lysis or ectopic colonization of periodontal pathogens around blood vessels can cause chronic inflammation. The long-term chronic inflammation of the vascular microenvironment is obviously beneficial to the formation of AS. Early studies found that PGN induced the production of pro-inflammatory cytokines through TLR2 and increased the vulnerability of AS plaques (Nijhuis et al., 2004). While the vascular endothelial dysfunction appeared in rats modeled by surgery and a high-cholesterol diet, the concentration of serum PGN was significantly increased (Tsunooka et al., 2005). It has been confirmed that TLR2 is expressed in macrophages in AS lesions. PGN activated monocytes to overexpress intercellular adhesion molecule-1 (ICAM-1) through the TLR2 and NF-κB pathways, promoting monocyte adhesion and chemotaxis to vascular diseases (Nijhuis et al., 2007; Xie et al., 2016). Using PGN to stimulate human coronary artery endothelial cells that were knocked out TLR2 through CRISPR-Cas9 technology, the expression levels of ICAM-1, IL-6, and IL-8 were significantly down-regulated (Wang et al., 2018). PGN can also be recognized by PGN recognition protein-1 (PGLYRP-1) in the innate immune system. The level of circulating PGLYRP-1 is associated with AS, coronary artery calcification, thickening of the abdominal aorta, and acute coronary syndrome (Brownell et al., 2016; Han et al., 2021). PGLYRP1 may promote the formation of AS plaques by regulating the overexpression of adhesion molecules in endothelial cells (Jin et al., 2021). PGN also triggers the up-regulation of vascular cell adhesion molecule-1 (VCAM-1) through the NOD1-RIP2-NF-κB axis, promotes the recruitment of myeloid cells, and leads to endothelial dysfunction (Gonzalez-Ramos et al., 2019).

### 2.2 Representative Periodontitis-Related DAMPs

#### 2.2.1 Neutrophil Extracellular Traps

Neutrophil extracellular traps (NETs) are an extracellular fibrous network structure produced by neutrophils after being stimulated, mainly composed of chromatin and cellular proteins (Brinkmann et al., 2004). The process of neutrophils forming NETs is called NETosis, including suicidal NETosis and survival vital NETosis, which is considered a cell death program different from apoptosis and necrosis (Yipp and Kubes, 2013). NETs recognize, trap, and restrict the spread of bacteria and other pathogens and highly express antimicrobial peptides and other antibacterial ingredients to delete pathogens ultimately (Papayannopoulos, 2018).

Numerous clinical studies have confirmed that NETs are closely related to the progression of PD. NETs expression in the inflamed gingival tissue was higher than that in the healthy control group (White P. C. et al., 2016). The expression of NETs in the gingival tissue of patients with periodontitis was higher than that of patients with gingivitis, indicating that the level of NETs is related to the severity of periodontal inflammation (Magan-Fernandez et al., 2019). The gingival biopsy samples of patients with PD and the purulent exudate in the periodontal pockets (Vitkov et al., 2009) found high expression of NETs, showing a fibrous network structure. There are many bacteria in the NETs and their mechanical entanglement, and they are closely arranged on the surface of the epithelium. A case-control study (Kaneko et al., 2018) found that the NETs level was positively correlated with the average probing depth and clinical attachment loss in patients with PD. NETs were detected in supragingival plaque biofilms, and NETs-related protein Myeloperoxidase (MPO) was found in saliva and biofilms, which confirmed that oral bacteria isolated from plaque biofilms could stimulate the formation of NETs (Hirschfeld et al., 2015). Neutrophils in PD are recruited by fibrin through myeloid integrin α_m_β_2_-binding motif and activated to generate NETs (Silva et al., 2021). Studies have found that *P. gingivalis* (Jayaprakash et al., 2015) and *Fusobacterium nucleatum* (*F. nucleatum*) (White et al., 2014) can stimulate neutrophils to produce reactive oxygen species (ROS). *Streptococcus sanguis* also increased the level of NETs marker citH3 and up-regulated the level of MPO (Oveisi et al., 2019). In summary, specific periodontal pathogens can stimulate neutrophils to produce ROS and release NETs.

Multivariate logistic regression analysis showed that the peripheral blood NET level was significantly positively associated with moderate to severe PD (Kaneko et al., 2018). Degradation of NETs in plasma is increased after periodontal therapy in PD patients (Moonen et al., 2020). Periodontal pathogens and excessive NETs produced during PD may participate in the progression of AS after entering the circulation. After entering the bloodstream, *P. gingivalis* binds to erythrocytes to avoid ROS destruction, thereby further activating the Rho GTPase signaling pathway, up-regulating CD11b/CD18, and promoting the activation of neutrophils (Borgeson et al., 2011; Damgaard et al., 2017). Periodontal pathogen DNA can be detected in carotid plaque. *T. forsythia* is significantly related to intraplaque hemorrhage and neutrophil activation, reflected in the increased release of MPO, cell-free DNA, and NETs (Range et al., 2014). The systemic inflammatory state caused by PD promotes the adhesion of neutrophils and endothelial cells by increasing oxidative stress parameters (superoxide and mitochondrial membrane potential) (Martinez-Herrera et al., 2018). However, studies are still limited to the relationship between periodontal NETs and AS. The differences between the histone composition and DNA sequence of periodontal NETs and other systemic inflammatory NETs, as well as the mechanism of periodontal NETs in AS plaque formation, deserve more efforts to reveal both *in vivo* and *in vitro*.

#### 2.2.2 High Mobility Group Box 1

High mobility group Box 1 (HMGB1) is a non-histone chromosome binding protein widely distributed in the nucleus of various cells. It plays an important role in stabilizing the structure of nucleosomes, regulating transcription factors, and DNA replication repair (Vijayakumar et al., 2019). HMGB1 can be released by necrotic or ruptured cells and activated immune cells (Andersson and Tracey, 2011). High levels of HMGB1 can be detected in the gingival crevicular fluid of patients with moderate to severe chronic PD (Luo et al., 2011; Paknejad et al., 2016), and it was positively correlated with periodontal clinical parameters (including plaque index, bleeding index, probing depth, and clinical attachment level) (Yu et al., 2019). Human gingival epithelial cells can increase the secretion of HMGB1 under the stimulation of TNF-α (Morimoto et al., 2008). Similarly, IL-1β promoted the secretion of HMGB1 in fibroblasts (Ito et al., 2012). Under the constant stimulation of periodontal infection, tissue cells continuously secrete HMGB1, which causes macrophages to be further activated and secrete cytokines, and amplifying the destruction of inflammation. This process can be inhibited by HMGB1 antibody (Yoshihara-Hirata et al., 2018). Excessive HMGB1 secreted by periodontal cells in PD or released by apoptosis can enter the circulation through osmosis and transmit the damage signal to the artery.

In AS, HMGB1 plays an important role. The increase of HMGB1 level directly leads to the mass production of cytokines, including IFN-γ, TNF-α, IL-1β, and IL-6, which promotes the formation of AS and reduces the stability of plaque (Su et al., 2015). In the AS model of rabbits, administration of HMGB1 and TNF-α can significantly aggravate the inflammation of advanced plaques (Kim J.-S. et al., 2016). In the ApoE^−/-^ mouse AS model induced by the Western diet, anti-HMGB1 antibodies were more than six times higher than regular diet ApoE^−/-^ mice and ApoE^+/+^ mice, indicating that HMGB1 autoimmunity is involved in the progression of AS (Pan et al., 2016). It was speculated that HMGB1 produced by PD might be involved in systemic diseases by acting on monocytes, macrophages and vascular endothelial cells (Morimoto-Yamashita et al., 2012). Bioinformatics analysis shows that HMGB1 is a potential molecular mechanism of the association between PD and AS (Ning et al., 2021). There have been reports that *P. gingivalis* elevated HMGB1 levels after myocardial infarction in mice (Srisuwantha et al., 2017). In addition, the circular RNA PPP1CC of *P. gingivali* can regulate the apoptosis of vascular smooth muscle cells through the HMGB1/TLR9/AIM2 axis (Liu J. et al., 2021). The role of HMGB1 produced by PD in the AS model remains to be discovered.

#### 2.2.3 Alarmins

S100 protein is a group of calcium-binding proteins. Its family has more than 20 members and participates in the metabolism of the cytoskeleton under physiological conditions (Vogl et al., 2014). When cells are damaged or phagocytes are activated, macrophages secrete S100A8, S100A9, and S100A12 (Foell et al., 2007). These proteins combine with PRRs as “alarmins”, activating immune cells and endothelial cells to promote inflammation (Goyette and Geczy, 2011). The levels of S100A8, S100A9, and S100A12 in saliva and gingival crevicular fluid of patients with PD were significantly increased (Kojima et al., 2000; Shin et al., 2019; Lira-Junior et al., 2020; Jimenez et al., 2021). Microbial infection can cause abnormal expression of S100 protein in gingival tissues and destroy the epithelial barrier function (Nishii et al., 2013). Immunohistochemistry and RNA sequencing showed that S100A8 and S100A9 were highly expressed in the ligature-induced PD (Maekawa et al., 2019). After mouse osteocyte-like cells (MLO-Y4-A2) were treated with S100A9, the expression of IL-6 and RANKL increased, and the p38/ERK/STAT3 signaling pathway was activated, indicating that alarmins were involved in periodontal bone destruction (Takagi et al., 2020). It is worth noting that the levels of S100A12 and C-reactive protein in the gingival crevicular fluid and serum of patients with PD are elevated and positively correlated with periodontal parameters (Pradeep et al., 2014), suggesting that alarmins may be the link between PD and AS.

The expression of S100A9 and SMemb protein (a phenotypic marker of smooth muscle cell proliferation) increased in aneurysm specimens from patients infected with *P. gingivalis* (Hokamura et al., 2010). This phenotype has been verified in mice. Studies have also found that *P. gingivalis* infection can up-regulate the expression of S100A9 in human aortic smooth muscle cells (hAOSMC), which makes hAOSMC change from a contractile to proliferative phenotype (Inaba et al., 2009). These may lead to a potential mechanism for PD to promote aortic intimal hyperplasia. Therefore, PD can accelerate AS through alarmins.

## 3 Periodontitis-Related PAMPs and DAMPs Recognition Receptors Exist in Atherosclerosis

### 3.1 Toll-like Receptors

Toll-like receptors (TLRs) are a type of PRRs that exist on the cell surface or endosome/lysosome membrane (Minton, 2019). So far, 10 TLRs have been found in humans. TLRs are mainly divided into two categories. TLR1, TLR2, TLR4, TLR5, TLR6, and TLR10 significantly recognize lipids and proteins, while TLR3, TLR7, TLR8, and TLR9 mainly recognize nucleic acids (Fitzgerald and Kagan, 2020). TLR1, TLR2, TLR4, TLR7, and TLR9 are highly expressed in the gingival tissue of patients with PD (Becerik et al., 2011; Scheres et al., 2011; Duarte et al., 2012; Ribeiro et al., 2012; Beklen et al., 2014; Chen et al., 2014). Under the infection of periodontal pathogens (such as *P. gingivalis*), LPS, flagella, and CpG DNA, etc., stimulate the corresponding TLRs, activate excessive innate immunity, and destroy periodontal tissue. These molecular patterns transfer chronic inflammatory signals from the oral cavity to cardiovascular tissues by releasing them into the blood or ectopic bacterial colonization, driving the activation of innate immunity in the vascular microenvironment and participating in the progression of AS.

The high degree of conservation of TLRs determines that TLRs closely related to PD are widely distributed in blood vessels and surrounding immune cells. Macrophages play a vital role in the pathology of AS. Under *P. gingivalis* stimulation, macrophages recognized LPS and flagella through TLR2 and TLR4, and CpG DNA through TLR9, secreted more IL-1β, IL-6, TNF-α, and adhesion molecules, and formed foam cells and participated in plaque formation (Gibson and Genco, 2007; Brown et al., 2015; Crump and Sahingur, 2016). At the same time, cholesterol crystals in blood vessels will amplify the activation of TLR2 and TLR4 signaling pathways of monocytes stimulated by *P. gingivalis*, and the mechanism may be related to the NLRP3 inflammasome (Kollgaard et al., 2017). Interestingly, mice lacking TLR2 and TLR4 have significantly reduced alveolar bone resorption compared with the control group under the condition of periodontal red-complex infection, and the serum oxidized low-density lipoprotein, nitric oxide, and lipid fractions levels were not altered. The AS lesions of the aortic arch in the experimental group also did not aggravate (Chukkapalli et al., 2017). After TLR9-deficient mice were stimulated with CpG DNA, the activation of NF-κB was down-regulated compared with wild-type, and the contractility of cardiomyocytes was increased (Knuefermann et al., 2008). Activating TLR9 in endothelial cells can promote neutrophil chemotaxis and vascular inflammation (El Kebir et al., 2009).

### 3.2 NOD-like Receptors

NLRs are similar to TLRs in that they are both signal transduction pattern recognition receptors. 23 NLR family members have been found in humans, including NOD1, NOD2, and NLRP3, etc (Zhen and Zhang, 2019). NOD1 and NOD2 are the first two NLRs discovered. They contain an N-terminal caspase recruitment domain and a C-terminal leucine-rich repeat sequence (Kim Y. K. et al., 2016). In healthy gingival tissue, NOD1 and NOD2 are more abundant than TLRs. Both *P. gingivalis* and *F. nucleatum* infection can induce high expression of NOD1 and NOD2 in periodontal tissues (Liu et al., 2014; Alyami et al., 2019). After NOD1 and NOD2 are activated, they recruit a series of downstream molecules and activate NF-κB and MAPK pathways (Jeon et al., 2012). PD aggravates vascular endothelial dysfunction through NOD1 and NOD2. *P. gingivalis* can stimulate the activation of endothelial cells and promote the up-regulation of E-selectin, NOD1, NOD2, and TLR2. This change depends on the NF-κB/p38/MAPK pathway. The use of small interfering RNA targeting NOD1 can suppress related signals (Wan et al., 2015). Compared with cells treated with NOD1 and NOD2 ligand stimulants, *P. gingivalis*-infected endothelial cells showed rapid lysis of receptor-interacting protein 1 (RIPK1) and RIPK2, suggesting that tumor necrosis factor receptor-1 (TNF-R1)-induced cell activation or death was involved in the invasion of arteries by periodontal pathogens (Madrigal et al., 2012).

NOD-like receptor protein 3 (NLRP3) NLRP3 is an essential member of the NOD-like receptor family. Its inflammasome complex consists of NLRP3, apoptosis-associated speck-like protein (ASC) containing a caspase recruitment domain (CARD) and pro-cysteinyl aspartate specific proteinase-1 (pro-caspase-1) composition. NLRP3 is encoded by autoinflammatory syndrome 1 (CIAS1) and has an N-terminal pyrin domain (PYD), a central nucleoside triphosphatase domain (NACTH domain), and a C-terminal leucine-rich repeat (LRR). When NLRP3 senses a danger signal, it interacts with the PYD of ASC. Then ASC recruits pro-caspase-1 through the same CARD and then aggregates it into NLRP3 inflammasomes. The activated inflammasomes prompt ASC to cleave pro-caspase-1 into active caspase-1, which promotes the maturation of IL-1β and IL-18, and induces inflammation and cell death (Haneklaus and O'Neill, 2015; Shao et al., 2015).

NLRP3 is a crucial mediator of periodontal infections involved in AS. The latest clinical study showed that NLRP3 was positively correlated with periodontal parameters, and periodontal treatment can effectively reduce the level of NLRP3 in gingival crevicular fluid (Shahbeik et al., 2021). The detection of high levels of NLPR3, ASC and IL-1β in saliva reflected the severity of periodontal inflammation (Isaza-Guzman et al., 2017). Further *in vivo* (Yamaguchi et al., 2017) and *in vitro* (Lu et al., 2017; Lian et al., 2018; Zhang et al., 2021) experiments proved that NLRP3 was involved in the regulation of periodontal inflammation. The saliva and serum levels of NLRP3 in patients with PD were elevated, suggesting that NLRP3 may be a mediator of periodontal infection and systemic diseases (Isola et al., 2021). In a clinical trial of 90 subjects, the level of NLRP3 in the serum of patients with coronary heart disease was significantly higher than that in the control group. It was positively correlated with the levels of serum IL-1β and IL-18 (Satoh et al., 2014). A study of 22 patients with chronic PD showed that the relative expression levels of ASC, NLRP3, and caspase-1 mRNA in peripheral blood decreased after initial periodontal treatment (Higuchi et al., 2020). It was suggested that periodontal treatment might prevent AS by reversing the release of inflammasome from periodontal tissue to the cardiovascular system. Compared with KDP136 (gingipain null mutant) or KDP150 (FimA defective mutant), wild-type (WT) ApoE^−/-^ mice infected with pg showed loss of alveolar bone and increased AS plaque area, and periodontal macrophages secreted more IL -1β, IL-18, and TNF-α. The expression of NLPR3 mRNA in the gingival tissue and the aorta were increased (Yamaguchi et al., 2015). *P. gingivalis* can also act synergistically with cholesterol crystals to stimulate the NLRP3 inflammasome to promote the secretion of AS-promoting cytokines by monocytes (Kollgaard et al., 2017). However, a study showed that *P. gingivalis* LPS could stimulate the increase of NLRP3 levels in endothelial cells instead of *P. gingivalis* stimulation (Huck et al., 2015), which seems to contradict *in vivo* studies. Therefore, the mechanism of NLRP3 in periodontal inflammation-promoting AS remains to be explored.

## 4 Blocking PAMPs and DAMPs has the Potential to Inhibit the Systemic Effects Caused by Periodontitis

With the continuous progress of periodontal inflammation, PAMPs and DAMPs can be released into the circulation, promoting the development of systemic diseases such as AS, rheumatoid arthritis, and inflammatory bowel disease, etc. As mentioned above, periodontal treatment can decrease systemic inflammation, which can be explained as the control of oral infections reduces the microbial burden caused by the release of PAMPs. The healing and reconstruction of damaged tissues cut off the source of DAMPs release.

In order to antagonize infection and inflammation, there have been attempts to find or synthesize inhibitors targeting molecular patterns. For example, the ubiquitous 14-3-3 β/α-A protein in zebrafish embryos specifically neutralizes PGN and protects the early embryonic host from pathogenic attacks (Wang et al., 2021). The artificially synthesized monoclonal antibody 2E7 targeting on muramyl-L-alanyl-D-isoglutamine (a highly conserved domain of PGN), suppressed the development of autoimmune arthritis and experimental autoimmune encephalomyelitis in mice by blocking NOD2-related pathways (Huang et al., 2019). It is suggested that the specific neutralization of PAMPs has potential value in regulating inflammatory diseases.

Synthetic Anti-lipopolysaccharide Peptides (SALPs) as effective inhibitors of PAMPs have been used to treat bacterial infectious diseases. SALPs capture the negatively charged phosphate and carboxylate in the LPS head group through the positively charged N-terminal residue, and then the C-terminal region interacts with the non-polar hydrophobic interaction of the lipid A acyl chain portion (Correa et al., 2019). The binding affinity of SALPs and LPS surpasses that of LPS-binding protein (LBP), showing excellent antibacterial properties. SALPs have a significant inhibitory effect on LPS-induced TNF-α secretion in monocytes. SALPs can also neutralize LPS-induced shock *in vivo* and have little impact on healthy organs (Gutsmann et al., 2010). In the cecum ligation and puncture (CLP)-induced mice model, SALPs can improve the contractile function of cardiomyocytes and reduce cardiac dysfunction (Martin et al., 2015; Martin et al., 2016). This indicated that SALPs have the potential to block the activation of periodontal-related LPS on innate immunity in AS.

Specific molecular patterns neutralizer such as SALPs have two disadvantages. First, there are many types of innate immune activation receptors, each of which recognizes a specific molecular pattern. We cannot guarantee to know the exact molecular pattern of each disease. There may be many models involved in the inflammatory response of the same disease. Secondly, these molecular patterns may be interrelated. Blocking only one of the specific pathways may not effectively suppress the inflammatory response. Both PAMPs and DAMPs include nucleic acids, such as DNA fragments in NETs and CpG DNA. Deoxyribonuclease I (DNase I) is the first DNA hydrolase to be discovered. In physiological conditions, DNase I contributes to the digestion of food, apoptosis, and elimination of necrotic cells (Mannherz et al., 1995). DNase I has been used as a common neutralizing agent to disrupt NETs structure to prevent DAMPs from over-activating innate immunity, and has preliminary applications in cancer treatment (Cools-Lartigue et al., 2013; White P. et al., 2016). In the periodontal destruction caused by plasminogen deficiency, the use of DNase I to remove NETs recruited and activated by excessive fibrin can significantly reduce alveolar bone resorption (Silva et al., 2021). The level of plasma DNase I of PD patients was significantly lower than healthy controls (White P. et al., 2016), indicating that the use of DNase I may contribute to the balance of nucleic acid metabolism in the circulation. In socially defeated ApoE^−/-^ mice, the aggravation of arterial plaque area was wholly diminished by DNase I treatment (Yamamoto et al., 2018). Therefore, DNase I may block the pathway of PD acting on AS by hydrolyzing DNA and its complexes. However, how to define the usage and dosage in the course of treatment and its potential damage to normal tissues remain to be considered.

Nucleic acids are generally negatively charged in their natural state. NA-binding polymers (NABP) represented by PAMAM-G3 are typically used in non-viral gene delivery. Recently, NABP, as a positively charged nucleic acid scavenger, and has been used in various inflammatory diseases to block the excessive activation of innate immunity by molecular patterns (Lee et al., 2011). Compared with the soluble polycations (like PAMAM-G3), the improved NABP nanoparticles have better biological safety and NA scavenging capacity, and demonstrates an excellent therapeutic effect in rheumatoid arthritis (Liang et al., 2018; Peng et al., 2019), sepsis (Dawulieti et al., 2020; Liu F. et al., 2021), and inflammatory bowel disease (Shi et al., 2022). Therefore, it can be feasible to regulate inflammation through non-specific clearance of DAMPs and PAMPs. At present, the research and development of such materials are still in their infancy, and it is expected to be applied to PD and AS models in the future.

## 5 Conclusion and Perspectives

The chronic inflammatory state of PD is closely related to cardiovascular disease. LPS, PGN, and CpG DNA released by periodontal pathogen infection, NETs, HMGB1, and alarmins cast by periodontal tissue destruction can enter the circulation and participate in vasoconstriction, endothelial dysfunction and the transformation of macrophage to foam cells. These molecule patterns join in the development of AS and affect the occurrence of CVD through TLR, NLR and other innate immune signaling pathways. PD and cardiovascular disease are two high-prevalence diseases in humans. Studying the mechanism of action between the two has significant public health significance. In this review, PAMPs and DAMPs are discussed as a complex mechanism of PD affecting AS. This shows that chronic inflammation represented by innate immune activation plays an important role in connecting oral cavity and systemic diseases. Naturally, we will consider whether eliminating molecule patterns will block this process. Through specific and non-specific removal of PAMPs or DAMPs, there have been preliminary applications in the regulation of inflammation. Similar methods will have application prospects in studying the relationship between PD and AS in the future.
